# Viscoelastic Metal-in-Water Emulsion Gel via Host–Guest Bridging for Printed and Strain-Activated Stretchable Electrodes

**DOI:** 10.1021/acsnano.2c04299

**Published:** 2022-08-04

**Authors:** Qi Wang, Xinyi Ji, Xue Liu, Yang Liu, Jiajie Liang

**Affiliations:** † School of Materials Science and Engineering, National Institute for Advanced Materials, Nankai University, Tianjin 300350, P.R. China; ‡ Key Laboratory of Functional Polymer Materials of Ministry of Education, College of Chemistry, Nankai University, Tianjin 300350, P.R. China; § Tianjin Key Laboratory of Metal and Molecule-Based Material Chemistry and Collaborative Innovation Center of Chemical Science and Engineering (Tianjin), Nankai University, Tianjin 300350, P.R. China; ∥ College of Light Industry Science and Engineering, Tianjin University of Science and Technology, Tianjin 300457, P.R. China

**Keywords:** liquid metal, emulsion gel, host−guest polymer, printed electronics, stretchable electrodes

## Abstract

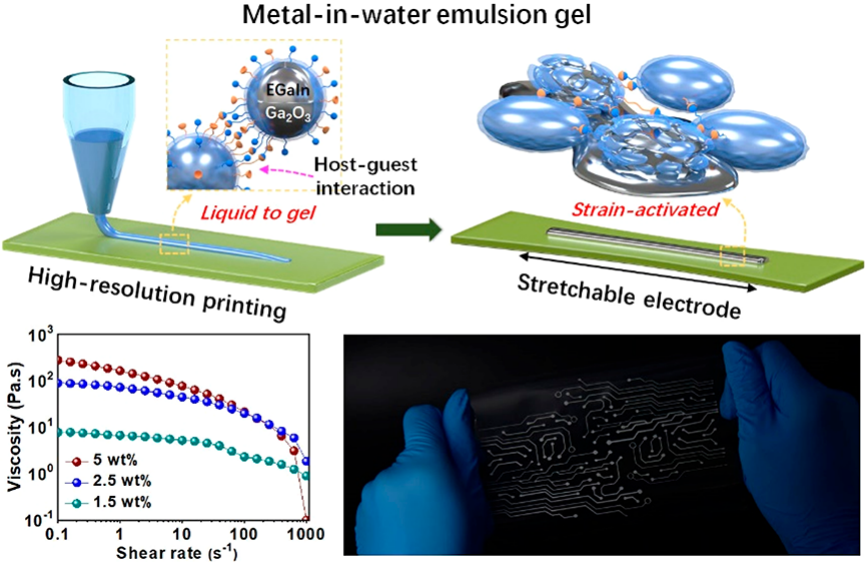

Stretchable conductive electrodes that can be made by printing technology with high resolution is desired for preparing wearable electronics. Printable inks composed of liquid metals are ideal candidates for these applications, but their practical applications are limited by their low stability, poor printability, and low conductivity. Here, thixotropic metal-in-water (M/W) emulsion gels (MWEGs) were designed and developed by stabilizing and bridging liquid metal droplets (LMDs) via a host–guest polymer. In the MWEGs, the hydrophilic main chain of the host–guest polymers emulsified and stabilized LMDs via coordination bonds. The grafted cyclodextrin and adamantane groups formed dynamic inclusion complexes to bridge two neighboring LMDs, leading to the formation of a dynamically cross-linked network of LMDs in the aqueous phase. The MWEGs exhibited viscoelastic and shear-thinning behavior, making them ideal for direct three-dimensional (3D) and screen printing with a high resolution (∼65 μm) to assemble complex patterns consisting of ∼95 wt % liquid metal. When stretching the printed patterns, strong host–guest interactions guaranteed that the entire droplet network was cross-linked, while the brittle oxide shell of the droplets ruptured, releasing the liquid metal core and allowing it to fuse into continuous conductive pathways under an ultralow critical strain (<1.5%). This strain-activated conductivity exceeded 15800 S/cm under a large strain of 800% and exhibited long-term cyclic stability and robustness.

## Introduction

Stretchable printed electronics have attracted attention in both the scientific and industrial communities.^1−7^ Rubbery stretchability is desired in various stretchable or wearable electronic applications,^8−15^ while printing technologies, with their highly versatile manufacturing, low environmental impact, and low costs, can help stretchable electronics penetrate broad consumer markets.^3,16^ A major barrier to creating stretchable printed electronics is the development of printable and stretchable conductors or electrodes.^3,6,7,17,18^ Liquid metals, such as nontoxic eutectic gallium indium (EGaIn), might be ideal candidates for printable and stretchable conductors because it is a fluid at room temperature (melting point of about 14.5 °C) and has a high metallic conductivity (34000 S/cm).^19−22^ Therefore, efforts to prepare stretchable conductors have focused on utilizing the fluidity and high surface tension (∼550 mN/m) of liquid metals by directly printing them into continuous conductive patterns with liquid-like conformability and deformability.^23−26^ However, the ultralow viscosity (2 mPa·s) and Newtonian flow behavior of liquid metals make it difficult to construct intricate patterns with high resolutions and output via direct printing technologies.^27,28^ As an alternative, printable composite inks formulated with core–shell structured liquid metal droplets (LMDs) and hydrophilic polymer solutions have been used to construct stretchable printed electrodes.^29−31^ Hydrophilic polymer additives, such as poly(vinyl alcohol) (PVA) and cellulose nanofibers (CNFs),^32^ tend to adhere to the solid Ga_2_O_3_ metal oxide shell of the LMDs, which stabilizes them and endow the inks with non-Newtonian properties. In the resulting printed patterns, the metal oxide shells can break via mechanical activation, and the encapsulated liquid EGaIn core flows out and fuses into a dynamic and stretchable conductive pathway. However, these composite inks suffer from at least one of the following restrictions: low conductivity, low maximum strain, low printing resolution, low viscosity, low stability, or poor dispersibility. Thus, it remains challenging to achieve printable liquid metal-based inks with a combination of high conductivity, stretchability and printability.^6,7,33,34^

Emulsion gels, which combine the properties of both viscoelastic gels and emulsions of one phase dispersed in another, provide a versatile and printable platform for preparing functional materials with tailored micro/nanostructures.^35,36^ The preparation of both oil-in-water (O/W) and water-in-oil (W/O) emulsion gels begins with producing emulsions using emulsifying agents, followed by introducing gelation agents to convert the emulsions into a gel-like state. This can be accomplished either by forming a cross-linked droplet network via interdroplet attractive interactions or by gelling of the continuous phase via interdroplet repulsive forces.^35−39^ Thus, the rheological properties, printability, formability, and functionality of the emulsion gels strongly depend on the interactions between neighboring droplets.^40^ Carefully regulating the interactions between droplets is an efficient way to realize printable emulsion gels with the desired properties.^41,42^ However, these approaches have been limited to only O/W or W/O emulsion gels. Liquid metals have a fluid state like water and oil, and it is envisioned that introducing proper emulsifying and gelling agents to control the interfacial interactions of LMDs might produce a emulsion gel system with the desired comprehensive properties.

Here, we introduced attractive interactions between neighboring LMDs to prepare a metal-in-water (M/W) emulsion gel (denoted as MWEG) by forming a dynamically cross-linked network of LMDs via a type of emulsifying and gelling agent of hydrophilic host–guest polymers. The LMDs were emulsified, covered, and stabilized using a hydrophilic host–guest polymer system that was based on a PVA main chain grafted with cyclodextrin (CD) and adamantane (AD) groups to provide strong dynamic host–guest interactions. The host–guest polymers bridged two neighboring LMDs via host–guest inclusion interactions to form a cross-linked network, resulting in gelation of the M/W emulsion. The shear-thinning viscoelastic behavior of the resulting MWEG enabled the extrusion-based three-dimensional (3D) printing and screen printing of MWEG into initially insulating patterns consisting of about 95 wt % LMDs with a resolution approaching 65 μm. Because the interdroplet host–guest inclusion interactions were much stronger than the breaking stress of the metal oxide shells of the LMDs, stretching ruptured the brittle metal oxide shells and released the liquid metal cores to form a continuous conductive pathway within the droplet network under an ultralow critical strain between 1 and 1.5%. This strain-activated electrode could be stretched by up to 800% strain while maintaining a conductivity over 15 800 S/cm and exhibiting stable and robust conductivity for more than 1000 stretch–release cycles to 200% strain. The MWEG is thus considered to present a versatile platform for the development of stretchable and printable conductive materials.

## Results and Discussion

**Figure 1:**
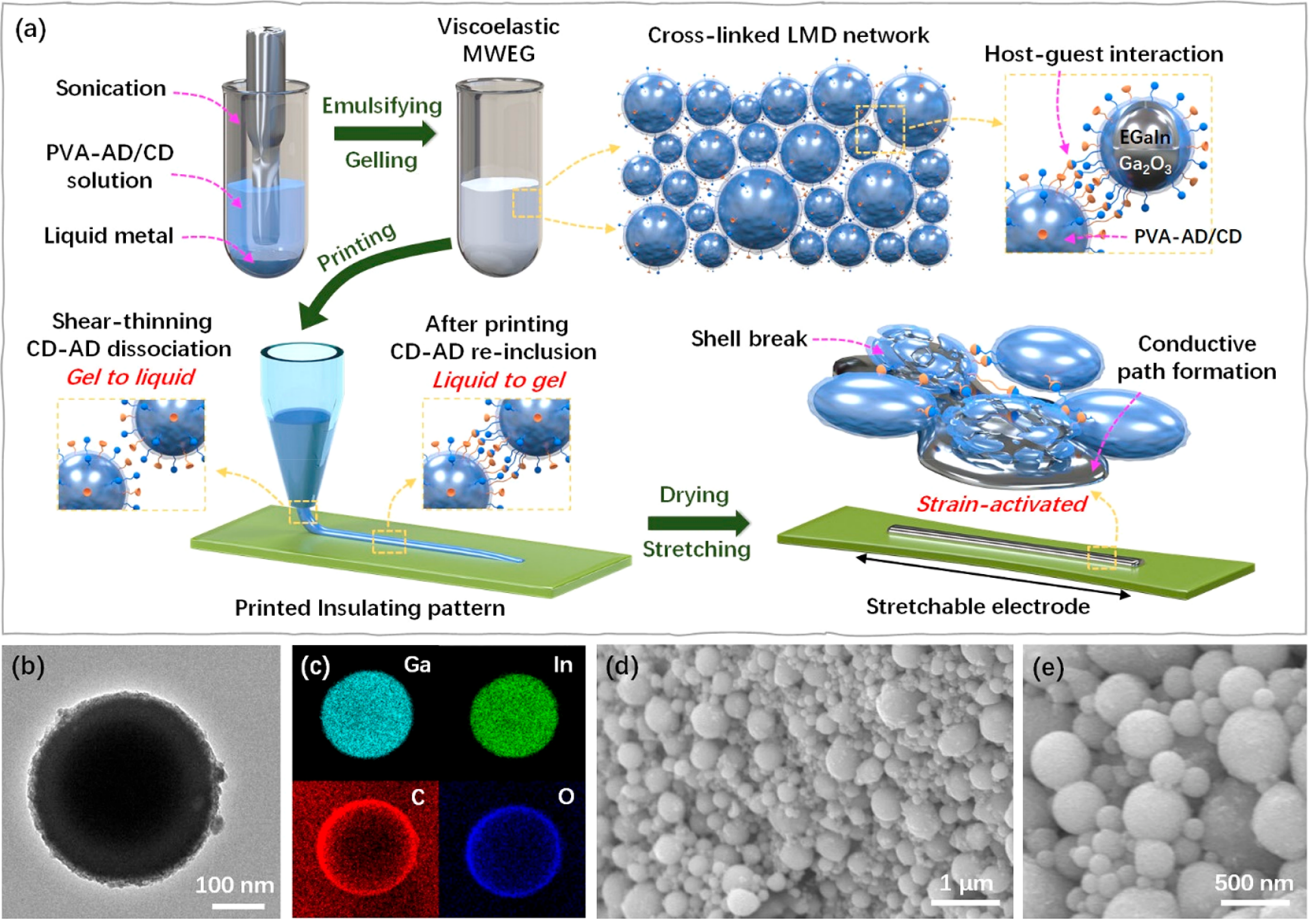
Fabrication of MWEG. (a) Schematic illustration of the procedure to prepare and 3D print an MWEG and strain-activated MWEG-based stretchable electrode. (b) TEM image and (c) EDS element maps (Ga, In, C, and O) of LMD wrapped by the hydrophilic host–guest polymers of PVA-AD and PVD-CD. C atoms (from PVA-AD and PVD-CD) were uniformly distributed around the surface of LMD. (d) SEM image and (e) magnified SEM image showing densely packed LMDs within the cross-linked MWEG network.

MWEGs were prepared by sonicating the liquid metal phase in the aqueous phase in the presence of emulsifying and stabilizing agents of PVA grafted with β-CD (labeled as PVA-CD) and adamantine (labeled as PVA-AD), as shown in Figures 1a and S1. During emulsification, uniform LMDs with typical core–shell structures were produced and stabilized in the aqueous phase using these hydrophilic host–guest polymers. Both PVA-AD and PVA-CD adhered to the Ga_2_O_3_ shell of LMDs via the coordination of hydroxyl groups from PVA with Ga^3+^ from a metal oxide skin.^32,34^ As shown in Figure 1b,c, a thin layer of the host–guest polymer with a thickness of about 10 nm uniformly wrapped an LMD. In contrast, no such thin polymer layer was observed on the LMD prepared by sonicating without added polymer stabilizer (Figure S2). As shown in Figure S3, LMDs were stabilized by the hydrophilic host–guest polymers in an aqueous solution under ambient conditions for 2 weeks without forming precipitates. In contrast, without the hydrophilic host–guest polymers, LMD precipitates could be clearly seen after storage for only 30 min. Because the AD can form stable inclusion complexes with β-CD with a high association constant (Figure S1),^43−45^ during subsequent gelling, PVA-AD and PVA-CD adhered onto LMDs bridged two neighboring LMDs via host–guest interactions to form a dynamic cross-linked LMD network in the aqueous phase, which facilitated gelation of the M/W emulsion (Figure 1a).

The scanning electron microscopy (SEM) images showed that PVA-AD and PVA-CD wrapped LMDs in a densely packed state within the cross-linked LMD network (Figure 1d,e). The size of the LMDs in MWEG was primarily determined by the sonication time during the emulsifying and gelling process (Figure S4). Unless noted otherwise, LMDs with an average diameter of 500 nm (Figure 1b) prepared by sonicating for 80 min were used in the following experiments. The presence of PVA-AD and PVA-CD greatly improved the size uniformity of LMDs after sonication (Figure 1d and S5). The size distribution of LMDs in MWEG was much narrower than that in CPI without the addition of a polymer stabilizer (Figure S5). Four formulations of MWEGs with mass ratios of LMDs: hydrophilic host–guest polymer (mole ratio of PVA-CD/PVD-AD = 1:1) and deionized water of 20:1.5:10 (denoted as MWEG-1.5, “1.5” representing the mass ratio of hydrophilic host–guest polymer), 20:1:10 (MWEG-1), 20:0.5:10 (MWEG-0.5), and 20:0.3:10 (MWEG-0.3) were prepared for subsequent studies. To evaluate the critical role of hydrophilic host–guest polymers on the emulsifying and gelling process, a series of controlled LMD composite inks with a mass ratio of LMDs/conventional polymer stabilizer/deionized water of 20:1:10 were prepared by replacing the hydrophilic host–guest polymer with PVA (labeled as CI-PVA, “CI” representing composite ink), poly(*N*-vinylpyrrolidone) (PVP, labeled as CI-PVP), cellulose nanofibers (labeled as CI-CNFs), and polyurethane (PU, labeled as CI-PU). The pure LMD composite ink (PCI) without polymer stabilizer was also prepared for comparison.

**Figure 2:**
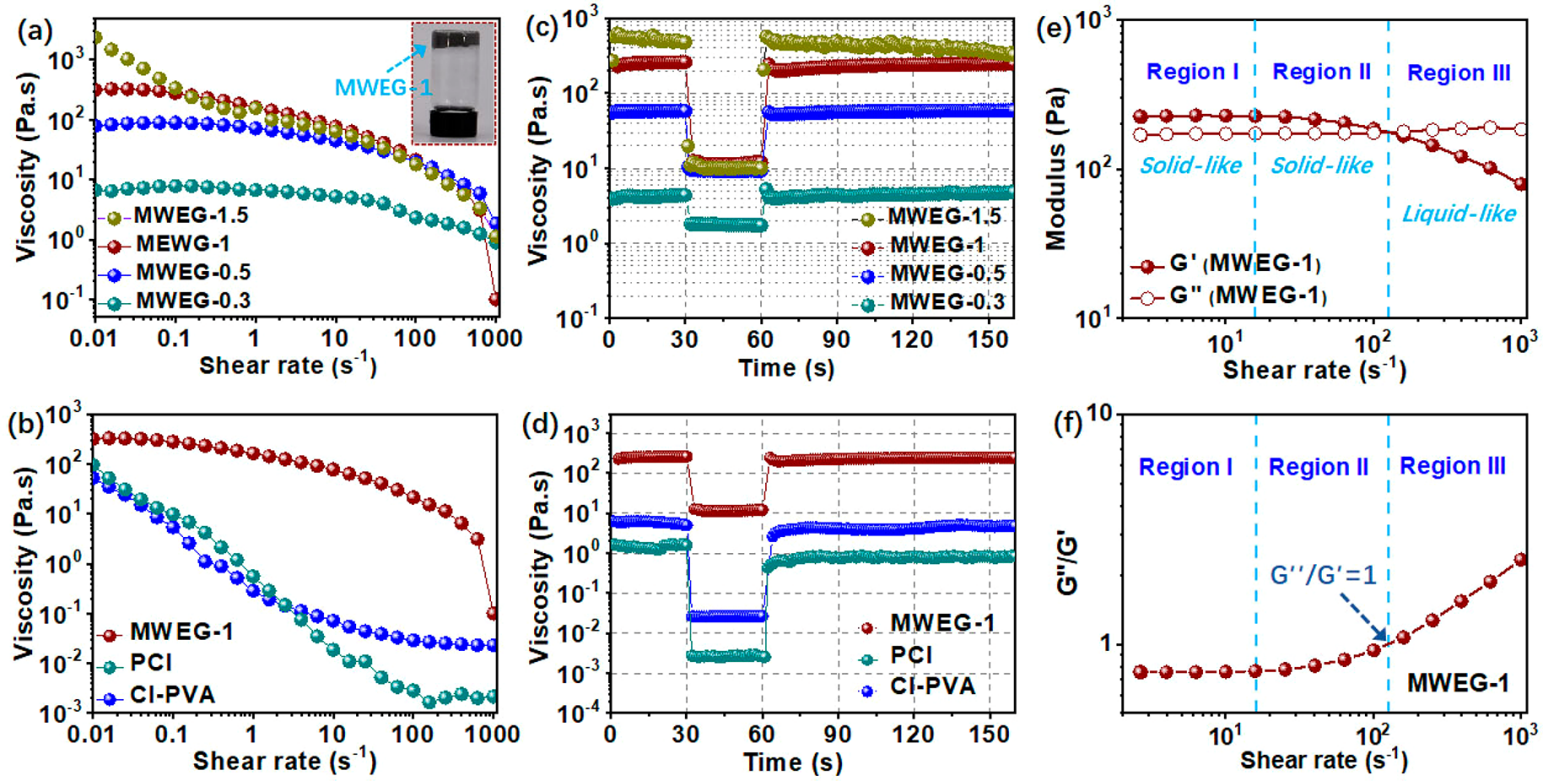
Viscoelastic property characterization of MWEGs. (a) Viscosity as a function of shear rate for MWEG-1.5, MWEG-1, MWEG-0.5, and MWEG-0.3. Inset shows the as-prepared gel-like MWEG-1. (b) Viscosity as a function of shear rate for MWEG-1, PCI, and CI-PVA. (c) Rheological behavior of MWEG-1.5, MWEG-1, MWEG-0.5, and MWEG-0.3 during simulated screen printing. (d) Rheological behavior of MWEG-1, PCI, and CI-PVA during simulated screen printing. (e) Variation of *G*′ and *G*″ for MWEG-1 as a function of shear rate. (f) *G*″/*G*′ ratio as a function of the shear rate for MWEG-1.

First, to understand how the contents of host–guest polymers influence the emulsion gel performance, a cone–plate rheometer was used to measure the viscoelastic and rheological properties of the MWEGs. As shown in Figure 2a, the curves of the viscosity as a function of shear rate for MWEG-1.5, MWEG-1, MWEG-0.5, and MWEG-0.3 all exhibited typical shear thinning behavior, as the fluid viscosity decreased upon increasing the stress. MWEGs with higher host–guest polymer contents showed a higher viscosity at the same shear rate. For instance, the viscosities of MWEG-1.5, MWEG-1, MWEG-0.5, and MWEG-0.3 at a shear rate of 0.01 s^–1^ were 2373.2, 317.2, 79.3, and 6.7 Pa·s, respectively. Thus, increasing the contents of the host–guest polymer from 0.99 wt % (MWEG-0.3) to 4.8 wt % (MWEG-1.5) transformed the diluent LMD emulsion suspension into a highly viscous emulsion gel (inset of Figure 2a). In contrast, although it also exhibited shear-thinning behavior, CI-PVA with 3.2 wt % PVA exhibited a much lower viscosity of 96 Pa·s at 0.01 s^–1^, which was close to that of PCI without polymer additive (52.2 Pa·s at 0.01 s^–1^) (Figure 2b). This indicates that the cross-linked LMD network formed via strong dynamic host–guest interactions between PVA-CD and PVA-AD played a vital role in improving the viscosity and facilitating the gelation of the M/W emulsion. In addition to a stabilizing agent, the host–guest polymers can serve as a gelling agent and thickener in the M/W emulsion system.

To evaluate the practical printability of MWEGs, a peak hold step (PHS) test in which the sample was held at different shear rates for three intervals was performed to simulate the extrusion-based 3D printing and screen-printing conditions. As shown in Figure 2c,d, the test samples remained in a high-viscosity state for 30 at 0.1 s^–1^ in the first interval. The shear rate was then suddenly increased to 200 s^–1^ and held for another 30 s to simulate extrusion and squeeze stroke. Finally, the shear rate was returned to 0.1 s^–1^ and held for 200 s to assess the viscosity recovery. The viscosities of MWEG-0.3, CPI, and CI-PVA in the first interval were all lower than 10 Pa·s, which was too low for 3D printing and screen printing, as it might decrease the pattern resolution. In contrast, MWEG-1 retained its viscosity in the first interval, and its viscosity decreased immediately from 227.6 to 11.3 Pa·s when entering the second interval. This sudden and large viscosity decrease was mainly attributed to the quick CD–AD dissociation from the host–guest polymer under a high shear force (Figure 1a), leading to the dynamic breakdown of the LMD network in the emulsion. After the shear rate was reduced back to 0.1 s^–1^, the viscosity of MWEG-1 quickly recovered to 201.5 and 217.4 Pa·s in only 10 s (at 70 s) and 20 s (at 80 s), corresponding to recovery rates of 88.5 and 95.5%, respectively (Figure 2c). Such a fast recovery time and high recovery rate were mainly attributed to the fast association kinetics of the CD–AD inclusion complexes after removing the shear force, which was conducive to the reformation of a cross-linked LMD network in the emulsion gel. This viscosity recovery was substantially superior to other viscoelastic inks based on conventional polymer thickeners (Figure S6).^3,28,46,47^ Notably, a fast recovery time and high recovery rate allowed for the quick leveling of MWEG-1 and uniform line formation after printing.^48,49^

To further study the viscoelastic behavior of MWEG-1, a stress sweep step (SSS) test was carried out, and the storage modulus (*G*′, elastic component, corresponding to solid-like behavior) and loss modulus (*G*″, viscous component, corresponding to liquid-like behavior) were measured as a function of the shear rate (Figure 2e).^3,48−50^Figure 2f shows the plots of tan δ (*G*″/*G*′) vs shear rate. The variation curves of the modulus and loss tangent for MWEG-1 could be divided into three distinct regions.^48−50^ Region I, also known as the linear viscoelastic (LVE) region, corresponded to the maximum deformation (or shear rate) that could be applied to the emulsion gels without breaking their network structures.^50^ In this region, the cross-lined LMD network in MWEG remained intact and elastically recovered under any applied stress or strain. Both the storage modulus and loss modulus of MWEG-1 were independent of the shear rate in region I. The ratio of liquid-like to solid-like behavior (*G*″/*G*′) within the LVE region was maintained at ∼0.75 (Figure 2f), indicating elastic-dominant (solid-like) behavior (*G*′ > *G*″). When entering region II, *G*′ decreased gradually, but *G*″ remained unchanged upon increasing the shear rate, while *G*′ remained higher than *G*″. This indicates the beginning of the dissociation of the CD–AD inclusion complexes and the gradual breakdown of the LMD network in the emulsion gel. Although MWEG-1 still exhibited elastic-dominated behavior (*G*′ > *G*″) in region II, the emulsion gel showed more viscous-like behavior upon increasing the shear rate.^50^ Region III began at the crossover point of *G*′ = *G*″, at which the value of *G*″ became higher than that of *G*′ as the shear rate increased. The LMD network was mostly destroyed within MWEG-1 in this region, and the emulsion behavior crossed over from solid-like behavior to liquid-like behavior, which was favorable for printing.^48,51^ In comparison, the viscoelastic behaviors of both MWEG-0.5 and MWEG-0.3 were viscous-dominated over the entire shear rate range (Figure S7), revealing incomplete LMD network formation within the emulsions due to too-low host–guest polymer contents.

**Figure 3:**
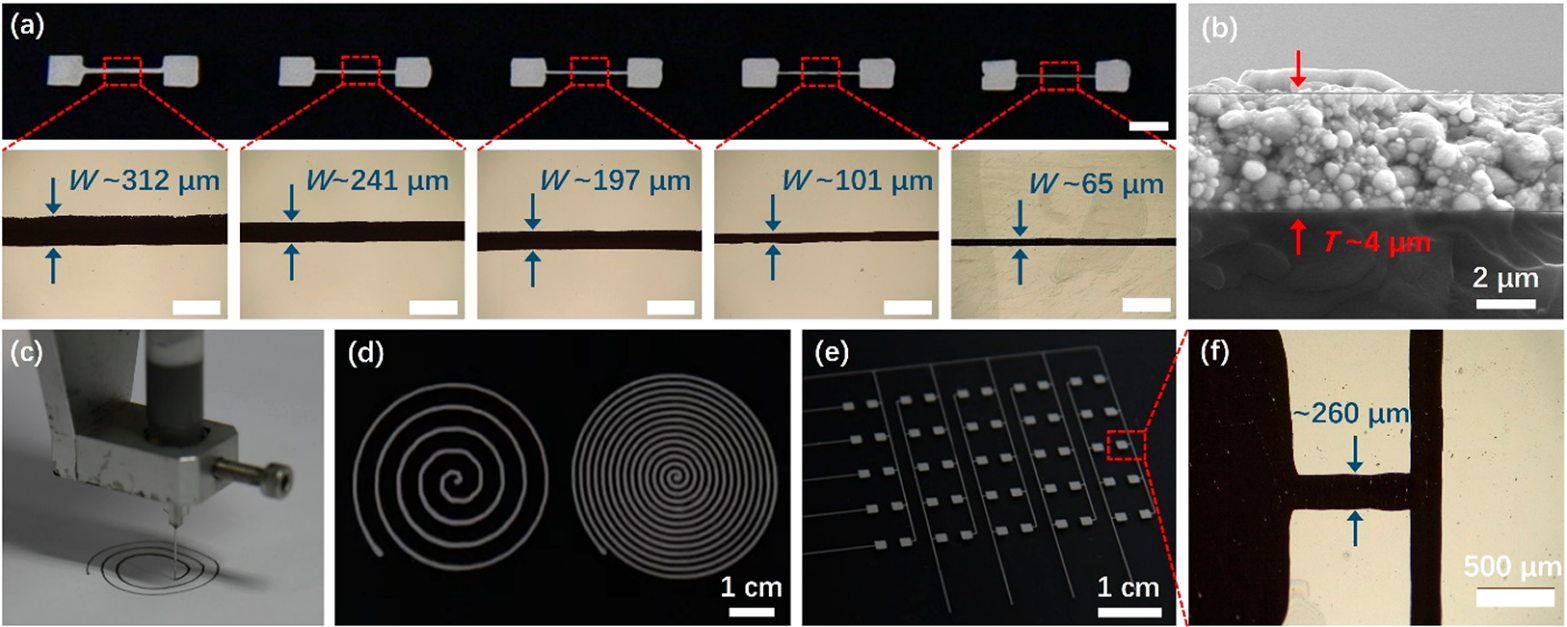
Printability of MWEG-1. (a) Optical images and the corresponding optical microscopy images of LMD lines screen-printed from MWEG-1 on a PU substrate. The printed line width (*W*) was about 65, 110, 215, 255, and 310 μm. Scale bars in the photograph and optical microscopy images are 2 mm and 500 μm, respectively. (b) SEM image of the cross section of screen-printed LMD line after drying. The thickness (*T*) of the printed line was about 4 μm. (c) 3D printing of MWEG-1. (d,e) Optical images of 3D-printed LMD lines and patterns. (f) Optical microscopy image of a 3D-printed LMD line with a width of about 260 μm.

The viscous gel-like state and rheological behavior enabled MWEG-1 to be screen-printed and 3D-printed into high-quality patterns. Because water was used as the only solvent in MWEG-1, the printed MWEG pattern could be dried under ambient conditions to obtain LMD patterns on various stretchable or flexible polymer substrates (Figure S8). Figure S8a shows a photograph of a series of LMD patterns screen-printed from MWEG-1 on a stretchable PU substrate. The optical images and optical microscopy images in Figure 3a further show the specific LMD lines with widths (*W*) of about 65, 101, 197, 241, and 312 μm, which were screen-printed from a stainless-steel stencil with line openings with widths of 50, 100, 200, 250, and 300 μm. All printed lines were uniform and continuous, even for the narrowest line with a width of 65 μm. The cross-sectional SEM image shows that the printed LMD line with an internal structure containing densely packed particles had a relatively uniform thickness of ∼4 μm (Figure 3b). These results reveal that the printing resolution of MWEG-1 can reach as high as 65 μm, which is much better than most previously reported printed patterns based on liquid metals (Table S1).^6,7,52−54^ In addition to screen printing, MWEG-1 could be extruded at a printing rate of 3 mm/s through a computer-controlled micronozzle (80 μm nozzle) and deposited onto flexible or stretchable substrates to construct complex 3D patterns (Figure 3c). Coiled microwires (Figure 3d) and a complete circuit pattern (Figure 3e) containing contact pads (size of 2 × 2 mm) and wirings (260 μm in width, Figure 3f) were deposited onto PU substrates using extrusion-based 3D printing. These results demonstrate the good printability of our emulsion gels, which might advance the manufacturing of stretchable devices based on liquid metals toward high-resolution fine patterning and scalable fabrication.

Owing to the presence of an insulating gallium oxide shell on the LMDs, the as-printed LMD patterns were initially nonconductive.^26,55^ Interestingly, when the LMD patterns printed from MWEG-1 (denoted as LMD-EG, containing about 95 wt % liquid metal) were uniaxially stretched, the LMDs within the cross-linked network were broken under tensile strain and could be transduced from strong CD–AD inclusion complexes of host–guest polymers into the brittle metal oxide skin of LMDs. This distinct strain-activation effect led to the outflow and subsequent fusion of liquid metal cores, resulting in the formation of stretchable conductive pathways within the cross-linked LMD electrodes (Figure 1a).^56^ Metal oxide skins were formed almost immediately on the surface of a liquid metal conductive pathway, preventing the leakage of liquid metals from the strain-activated electrodes.^56^

**Figure 4:**
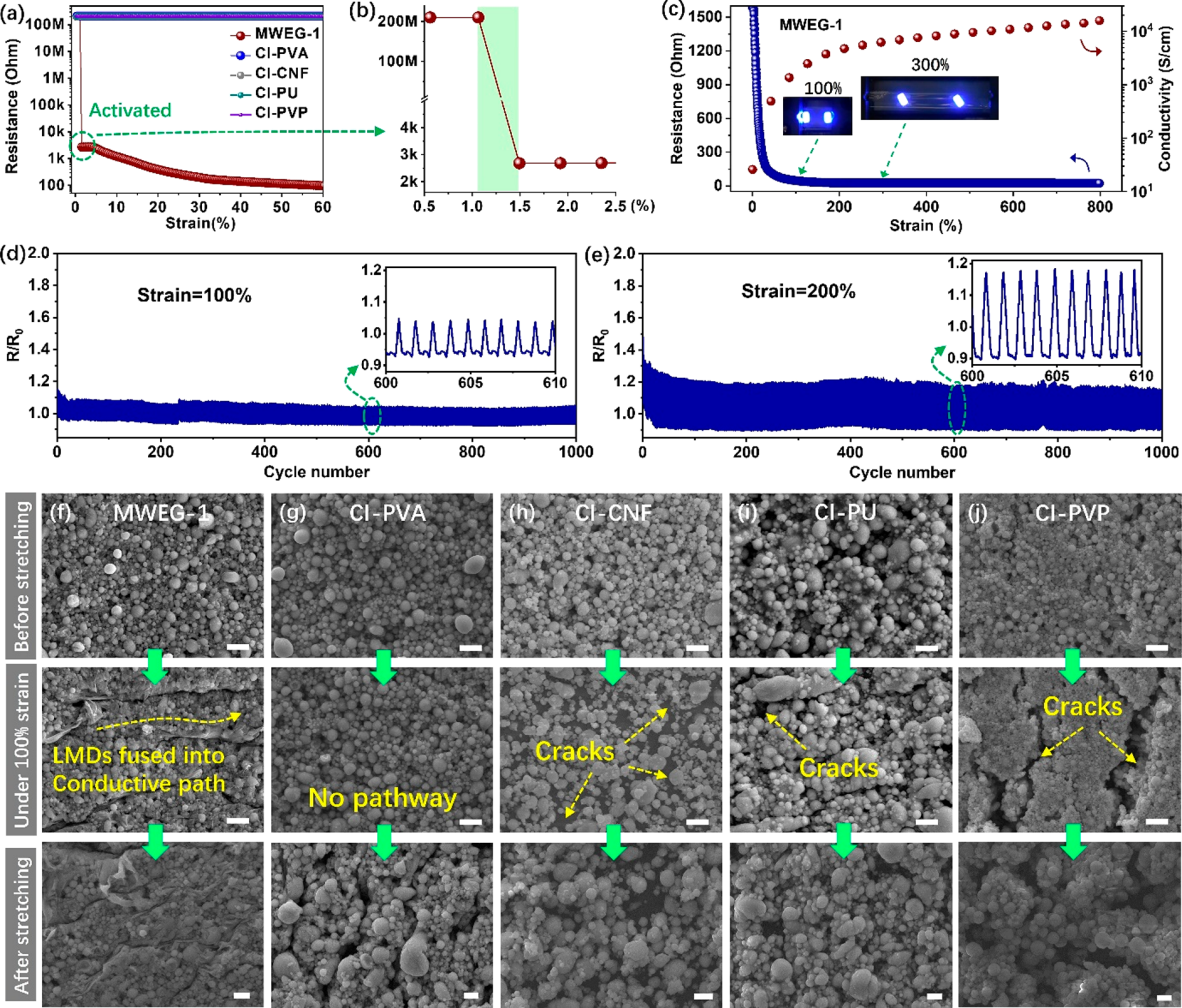
Strain-activated conductivity of LMD electrodes printed from MWEG-1. (a) Resistance vs the strain of LMD electrodes printed from MWEG-1, CI-PVA, CI-CNF, CI-PU, and CI-PVP. (b) Critical strain value of the LMD electrode printed from MWEG-1 was located between 1 and 1.5%. (c) Resistance changes (blue cycle) associated with electrical conductivity (red cycle) vs strain for the LMD electrodes printed from MWEG-1. Inset shows the lighting of LEDs connected to the LMD electrodes under 100 and 300% strain. Normalized resistance changes of the LMD electrodes printed from MWEG-1 subjected to (d) 100% strain and (e) 200% strain for 1000 stretch–release cycles. SEM images of LMD electrodes printed from (f) MWEG-1, (g) CI-PVA, (h) CI-CNF, (i) CI-PU, and (j) CI-PVP before stretching, under 100% strain, and after stretching. The scale bar is 1 μm.

**Figure 5:**
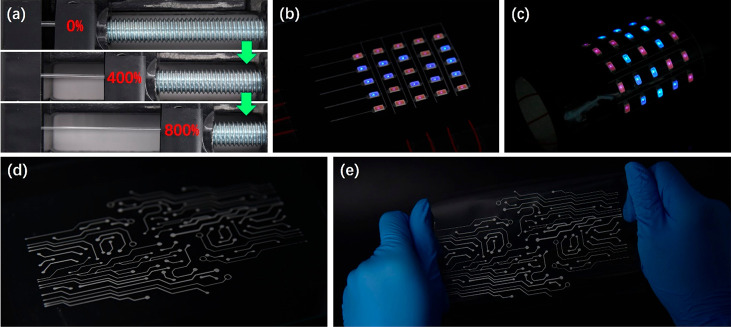
Demonstration of stretchable LMD electrodes printed from MWEG-1. (a) Photographs of LMD-EG stretched up to 800% strain. (b,c) Photographs of a stretchable LED display with 25 chips integrated with an LMD-EG circuit fabricated using extrusion-based 3D printing on a PU substrate. LMD-EG circuit activated by stretching to 50% strain. (d,e) Photographs showing a complex stretchable LMD-EG circuit pattern printed on a PU substrate.

The onset of conductivity as a function of strain during the stretching of LMD-EG is presented in the inset of Figure 4a. Strikingly, it showed a 5 orders of magnitude drop in resistance between 1 and 1.5% strain (Figure S9 and Movie 1), with a continuous and gradual decrease as the LMD-EG was further stretched to 800% strain (Figure 4c). Correspondingly, the calculated electrical conductivity reached as high as 15800 S/cm at 800% strain, which stands out among other reported stretchable conductors (Table S2).^26,57,58^ The failure at over 800% strain was attributed to the breakdown of the PU substrate rather than the breakdown of LMD-EG (Figure 5a). The applied strain played an important role in changing the conductivity of the printed electrodes, and a larger strain induced a higher conductivity in the electrodes after recovery. For instance, the conductivity of the LMD-EG released from 500 and 800% strain reached 13300 and 17200 S/cm, respectively. Moreover, as shown in Figure S10, printed lines with different line widths (from 300 to 100 μm) had similar critical strain values.

In contrast to LMD-EG, none of the LMD patterns made of CI-PVA, CI-CNF, CI-PU, and CI-PVP exhibited this strain-activated conductivity effect, even when their printed patterns were stretched to over 60% strain (Figure 4a). The strain-activated shell rupture and liquid core release to form a continuous conductive pathway could be observed in LMD-EG under tensile strain in the SEM image in Figure 4f. In comparison, microcracks, rather than the breakdown of the oxide shell, and the formation of conductive pathways appeared in the LMD patterns made of CI-PVA, CI-CNF, CI-PU, and CI-PVP under strain (Figure 4g–j). This indicates that the host–guest polymer played a critical role in causing a strain-activated conductivity effect on the LMD electrodes. According to previously published studies, the critical stress required to rupture the oxide shell of LMDs with an average diameter of 500 nm was calculated to be about 500 nN.^33^ The association constant (*K*) of AD and β-CD from the host–guest polymers in a deionized water environment at a neutral pH was about 10^4^ M^–1^.^59^ Accordingly, the Gibbs free energy (Δ*G*) was calculated to be 6.15 × 10^4^ J/mol according to the following equation:
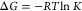
where *R* is the universal gas constant, and *T* is the thermodynamic temperature. The Δ*G* of each pair of AD and CD is represented by Δ*G*/*N*_A_, where *N*_A_ is Avogadro’s number. The height of CD and AD was about 7.9 and 6.36 Å,^60^ respectively, and the diameter of the circular truncated cone was about 15.4 Å. Considering the added amounts of host–guest polymers and the average diameter of LMDs, the pairs of AD and CD adhered to the surface of the LMDs were calculated to be 6.1 × 10^5^. The total Δ*G* of AD and CD wrapped around LMDs was thus calculated to be about 6.2 × 10^–13^ J, which was much higher than the critical shell rupture energy of 3.5 × 10^–15^ J calculated for the LMDs (supporting Note 1). Thus, when uniaxially stretching the LMD-EG, the LMDs were first broken under tensile strain, as the applied force could be transduced from the strong cross-linked network of the host–guest polymers to the brittle metal oxide skin of LMDs.

The stability and robustness of the strain-activated LMD-EG electrodes were also studied. The normalized resistance change (*R*/*R*_0_, where *R* is the resistance under specific strain, and *R*_0_ is the initial resistance) over 1000 cycles to 100% and 200% strain was measured, as shown in Figure 4d,e, respectively. Interestingly, the measured resistance showed a gradual drop in the first tens of strain cycles, which was mainly due to more droplet rupture and more conductive pathway formation during the initial cyclic strain activation. Then, the LMD-EG electrodes exhibited outstanding stability and robustness during subsequent cyclic stretch–release measurements, demonstrating good mechanical reliability for practical applications. Moreover, the conductivity of the LMD-EG electrodes was maintained over two months under ambient conditions.

The high printing resolution, high electrical conductivity, and ultrahigh stretchability of LMD-EG provide an outstanding platform for the construction of stretchable printed electronics. Figure 5b,c shows an example of a stretchable LED display with 25 chips integrated with the 3D-printed LMD-EG circuit. The printed circuits were activated by stretching, and all LEDs were operational. Importantly, the LEDs operated stably after the LMD-EG circuit was stretched to 50% strain over 1000 cycles (Figure S11). Figure 5d,e shows another example of a complex stretchable circuit that was fabricated using 3D printing combined with screen printing.

## Conclusions

In summary, a type of metal-in-water emulsion gel was designed and prepared by developing a hydrophilic host–guest polymer. The liquid metal droplets were emulsified and stabilized in water via coordination bonding, followed by gelling of the emulsion via the formation of a host–guest bridged network of liquid metal droplets in the continuous aqueous phase. The dynamic host–guest polymers, which simultaneously acted as the stabilizing agent, gelling agent, and thickener, endowed the metal-in-water emulsion gel with ideal viscoelastic properties and rheological behavior for the 3D printing and screen printing of complex patterns with a high resolution (∼65 μm) and output. Importantly, the printed patterns achieved strain-activated conductivity and an extremely low critical strain below 1.5%. The strain-activated electrodes exhibited a conductivity of over 15800 S/cm at a large strain of 800% and impressive cycling stability and robustness. It is envisioned that our metal-in-water emulsion gels will become a versatile printable platform for the construction of stretchable or wearable electronics with a high integration density, large deformability, multifunctionality, and high reliability.

## Experimental Section

### Raw Materials

PVA (average molecular weight of 205 000 g/mol) was provided by Aladdin Industrial Co., Ltd. 6-Monodeoxy-6-monoamino-β-CD (NH_2_-CD) was obtained from Shanghai D&B Biological Science and Technology Co., Ltd. Amantadine (NH_2_-AD), succinic anhydride, *p*-toluenesulfonic acid, *N*-(3-(dimethylamino)propyl)-*N*′-ethylcarbodiimide hydrochloride (EDC), and 1-hydroxybenzotriazole hydrate (HOBt) were purchased from Tianjin C&S Biochemical Technology Co., Ltd. and used as received without further purification. All other materials and solvents were of analytical reagent grade. EGaIn was fabricated by mixing melted gallium and indium (Ga/In = 75:25, by weight) overnight.

### Synthesis of PVA

First, 4 g of PVA was dissolved in 20 mL of dimethyl sulfoxide (DMSO) at 80 °C. Then 455 mg of succinic anhydride and 13 mg of *p*-toluenesulfonic acid were added to the solution as catalysts. The solution was maintained at 50 °C for 48 h under constant stirring. The impurities were removed by conducting dialysis for 5 days in deionized water. The dialyzed solution was freeze-dried to obtain PVA acid.

### Synthesis of PVA-AD

The samples of PVA-AD were prepared by a reaction between amantadine and the carboxylic acid groups of PVA acid. PVA acid (500 mg) was added to 10 mL of dimethylformamide (DMF) at 80 °C and stirred for 30 min until completely dissolved. Then 34 mg of NH_2_-AD, 56 mg of EDC, and 5 mg of HOBt were added to the PVA acid solution. The mixture was immersed in a preheated oil bath at 70 °C for 24 h under constant stirring. The reaction was cooled to room temperature and purified by dialysis for a week and then freeze-dried.

### Synthesis of PVA-CD

First, 500 mg of PVA acid was dissolved in 20 mL of DMF at 80 °C and stirred for 30 min. Then 254 mg of NH_2_-CD, 53 mg of EDC, and 5 mg of HOBt were added to the PVA acid solution. The mixture was maintained in a preheated oil bath at 50 °C for 24 h under constant stirring. The reaction was terminated by immersing the solution in an ice water bath. The crude product was purified by dialysis in deionized water for 5 days, followed by freeze-drying.

### Preparation of MWEGs and LMD-Based Composite Inks

Typically, bulk EGaIn (3.0 g) was added to an ethanol solution (30 mL) of different concentrations of PVA-CD/AD (PVA-CD and PVA-CD were mixed in a 1:1 weight ratio). The mixture was sonicated (BILON92-II; power of 500 W) in an ice–water bath with an optimal time (from 20 to 80 min). Free PVA-AD and PVA-CD in the dispersion were removed by centrifugation at 10000 rpm for 10 min. A 2000 mg mL^–1^ dispersion was prepared by dispersing the sonicated LMDs in deionized water and then agitating them using a VORTEX mixer at 1000 rpm for 60 min to obtain the MWPEGs. For comparison, three formulations of MWEGs with a mass ratio of LMDs, hydrophilic host–guest polymer (mole ratio of PVA-CD/PVD-AD = 1:1), and deionized water at 20:1:10 (MWEG-1), 20:0.5:10 (MWEG-0.5), and 20:0.3:10 (MWEG-0.3) were prepared for studies. Moreover, a series of controlled LMD composite inks with a mass ratio of LMDs, conventional polymer stabilizer, and deionized water of 20:1:10 were prepared by replacing the hydrophilic host–guest polymer with PVA (CI-PVA), PVP (CI-PVP), CNF (CI-CNF), and PU (CI-PU). PCI (with 2000 mg mL^–1^ of LMDs) without any polymer stabilizer.

### 3D Printing and Screen Printing of MWEGs

Conductive films were fabricated using screen printing and extrusion-based 3D printing. 3D printing was performed by a benchtop robot (FiSNAR F7304N) using a preprogrammed procedure. The ink extrusion was controlled by an air-powered fluid dispenser (FiSNAR, DC 100) with a needle diameter of 250 μm, a pressure of 1.1 bar, and a moving speed of 3 mm s^–1^. The screen-printing device was comprised of a rubber squeegee, a precision stainless-steel screen mesh (Dongguan XiangPeng Screen Printing Equipment Co., Ltd.), and a base plate. The screen mesh with an appropriate pattern was installed in the screen printer (TC-4060k screen printer purchased from Dongguan Ta Chen Screen Printing Machine & Materials Co., Ltd.) before printing. Following installation, the composite gel was applied onto the screen-printing plate and printed onto the PU substrate by sliding the squeegee over the stencil. The surface of the PU substrate was treated with O_3_ plasma to improve its hydrophilicity. The printing speed, printing force, and angle between the rubber squeegee and screen mesh were specifically optimized for the composite ink. The conductive film was obtained and then dried under ambient conditions for 3–5 min.

### Characterization

The surface morphology and structure were imaged using a field-emission SEM (JSM-7800, Japan). TEM images were captured using a transmission electron microscope (JEM-2800, Japan). XPS characterization was conducted using an ESCALAB 250Xi system (Thermo Scientific). Optical microscopy images and digital camera images of the samples were obtained using an upright metallurgical microscope (Leica DM750 M) and Canon 5D Mark III camera, respectively. The rheological behavior of the screen-printing inks was evaluated at 25 °C using a DHR-2 rheometer (TA Instruments) with a 20 mm parallel plate geometry and 900 μm gap. Before each test, a preconditioning step was applied at a shear rate of 0.1 s^–1^ for 10 s. The apparent viscosity of the inks was studied at shear rates from 0.1 to 1000 s^–1^. A PHS test was performed at constant shear rates in three intervals (0.1 s^–1^ shear rate for 30 s, 200 s^–1^ for 30 s, and 0.1 s^–1^ for 100 s) to simulate the screen-printing process. An SSS test was performed with an oscillation stress ranging from 1 to 1000 Pa at a frequency of 1 Hz. The resistance variation was measured using a Keithley 2400 system. Stretching tests and cyclic strain tests were performed using a motorized linear stage with a built-in controller (Zolix). Electrical conductivity was calculated using the equation σ = *L*/(*RS*), where *L* is the length of the tested sample, *R* is the sample resistance, and *S* is its cross-sectional area.

## Supporting Information Available

Comparison of electrical conductivity of stretchable conductors (MP4)
Molecular structure of PVA-AD and PVA-CD, TEM and SEM images of LMDs, stability of LMD mixtures, rheological behavior of CI-PVP, CI-CNF, and CI-PU, a series of screen-printed and 3D-printed LMD-EG patterns on various substrates (PDF)
