# Supplementary material for: Viscoelastic Metal-in-Water Emulsion Gel via Host–Guest Bridging for Printed and Strain-Activated Stretchable Electrodes
Source: ACS Nano. 2022 Aug 4;16(8):12677–85. doi: 10.1021/acsnano.2c04299 (PMC9413406; doi:10.1021/acsnano.2c04299)
Supplement: Supplementary file 2 — nn2c04299_si_002.pdf [file nn2c04299_si_002.pdf]

Supplementary Information

Viscoelastic metal-in-water emulsion gel via host-guest bridging for printed and strain-activated stretchable electrodes

Qi Wang[‡1], *Xinyi Ji*[*‡1], *Xue Liu*[1], *Yang Liu*[14], *Jiajie Liang*[*123]

[1] School of Materials Science and Engineering, National Institute for Advanced Materials Nankai University, Tianjin 300350, PR China. [2] Key Laboratory of Functional Polymer Materials of Ministry of Education, College of Chemistry, Nankai University, Tianjin 300350, PR China. [3] Tianjin Key Laboratory of Metal and Molecule-Based Material Chemistry and Collaborative Innovation Center of Chemical Science and Engineering (Tianjin), Nankai University, Tianjin 300350, PR China. [4] College of Light Industry Science and Engineering, Tianjin University of Science and Technology, Tianjin, 300457, China

Address correspondence to: xyji06@nankai.edu.cn, liang0909@nankai.edu.cn

[‡] Equal contribution

Supporting Figures and Tables

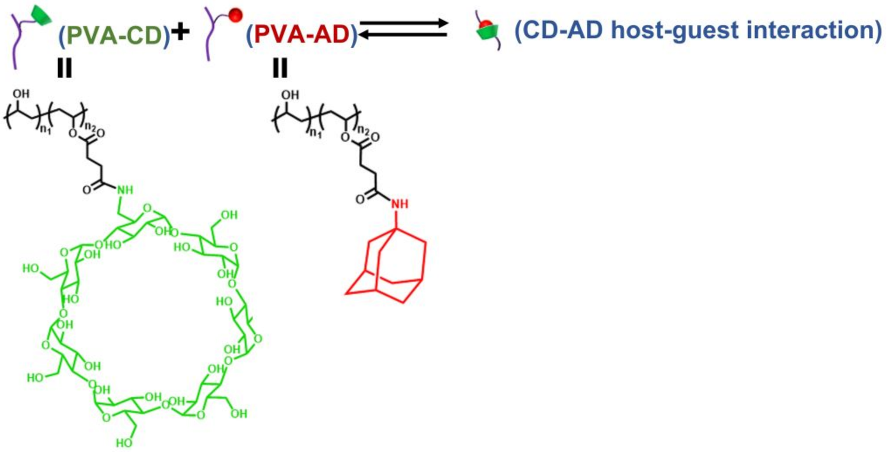

Figure S1. Molecular structure of PVA-AD and PVA-CD, and formation of CD-AD inclusion complexes.

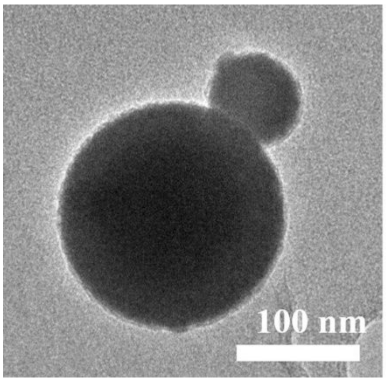

Figure S2. TEM image of pure liquid metal droplets. No thin polymer layer was observed on the surface of droplets.

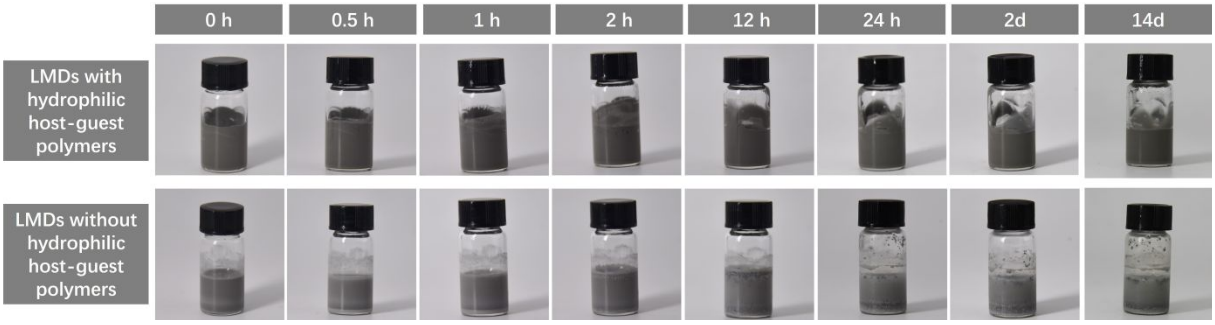

Figure S3. Images of aqueous mxitures of LMDs with and without hydrophilic host-guest polymers. The mixtures were stored in ambient conditions over two weeks.

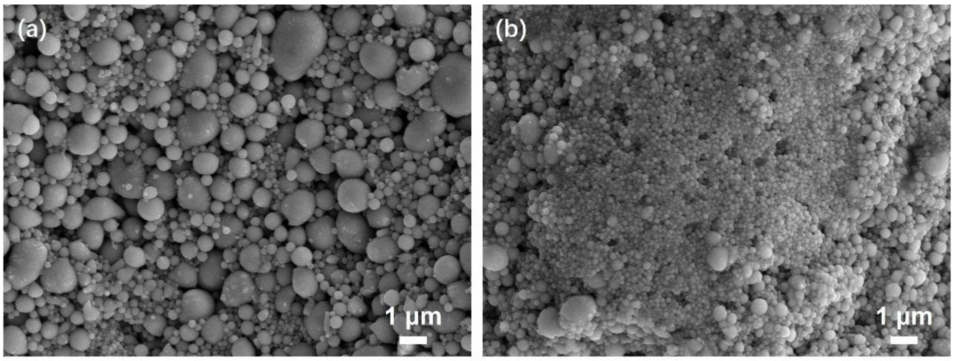

Figure S4. SEM images of LMDs prepared by sonicating in the presence of host-guest polymers for (a) 40 min and (b) 120 min. The average diameter of LMDs in is about 2 μm and 100 nm in (a) and (b), respectively.

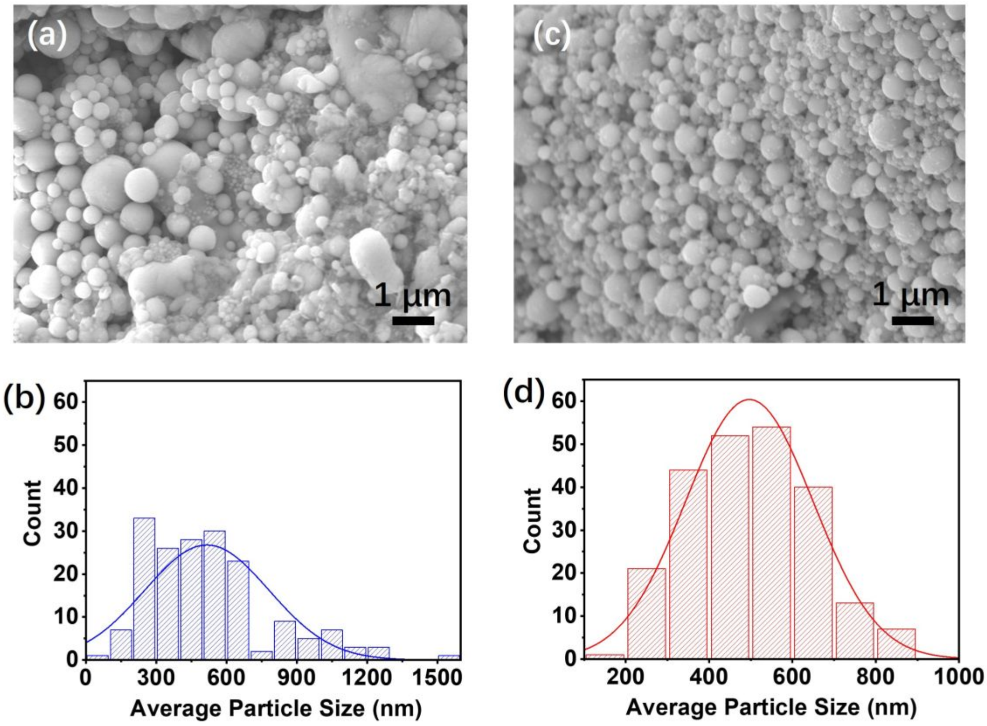

Figure S5. SEM images of (a) LMDs prepared by sonicating for 80 min without addition of any polymer stabilizer and (c) LMDs with PVA-CD and PVD-AD in MWEG. The plots of size distribution of (c) LMDs in (a) and (d) LMDs in (c).

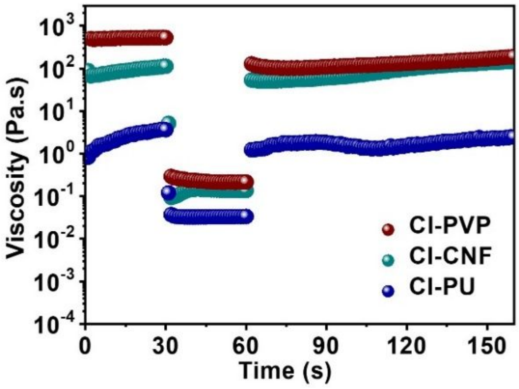

Figure S6. Rheological behavior of CI-PVP, CI-CNF, and CI-PU during simulated screen-printing process. The viscosity recovery rate is much lower than that of MWEG-1.

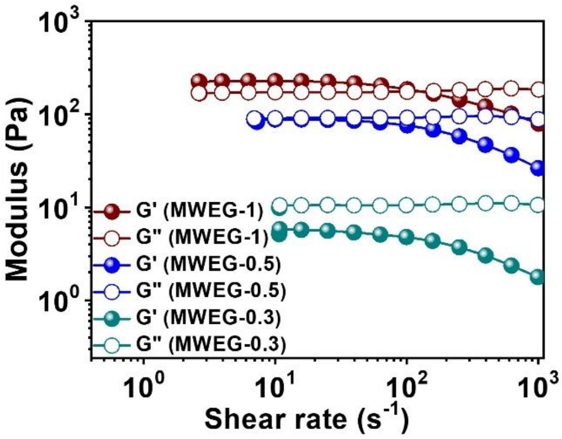

Figure S7. Variation of G´ and G for MWEG-1, MWEG-0.5, and MWEG-0.3 as a function of shear rate.

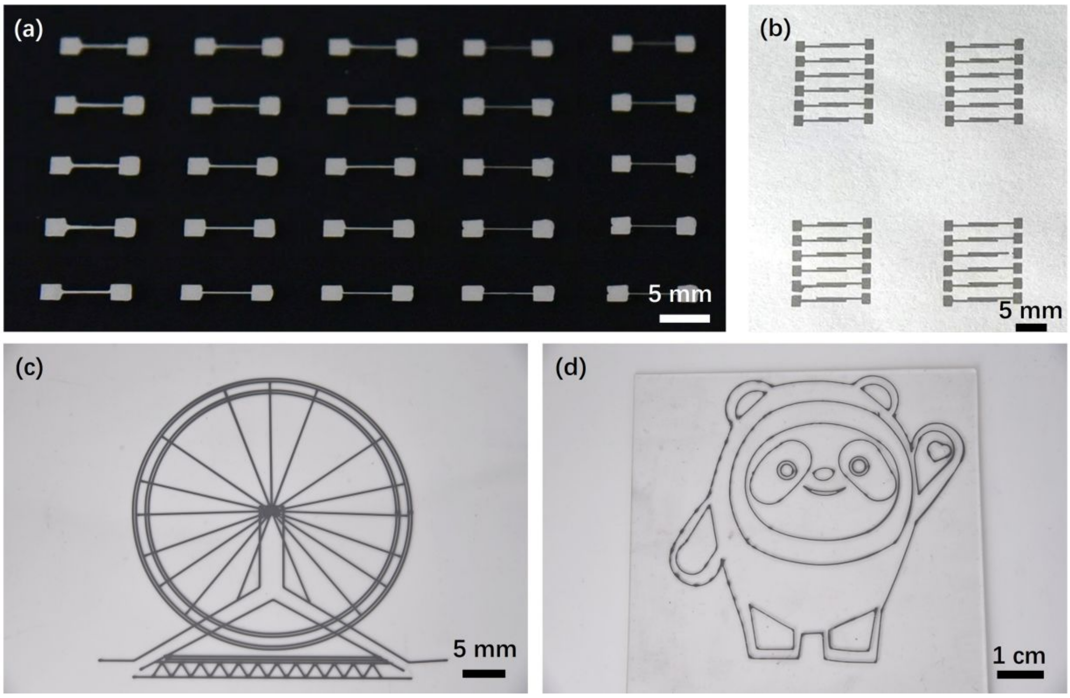

Figure S8. A series of screen-printed and 3D-printed LMD-EG patterns on various substrates. (a) LMD-EG patterns screen-printed on PU substrate. (b) LMD-EG interdigital electrodes screen-printed on paper substrate. (c) LMD-EG patterns 3D-printed on PET substrate. (d) LMD-EG patterns 3D-printed on PDMS substrate.

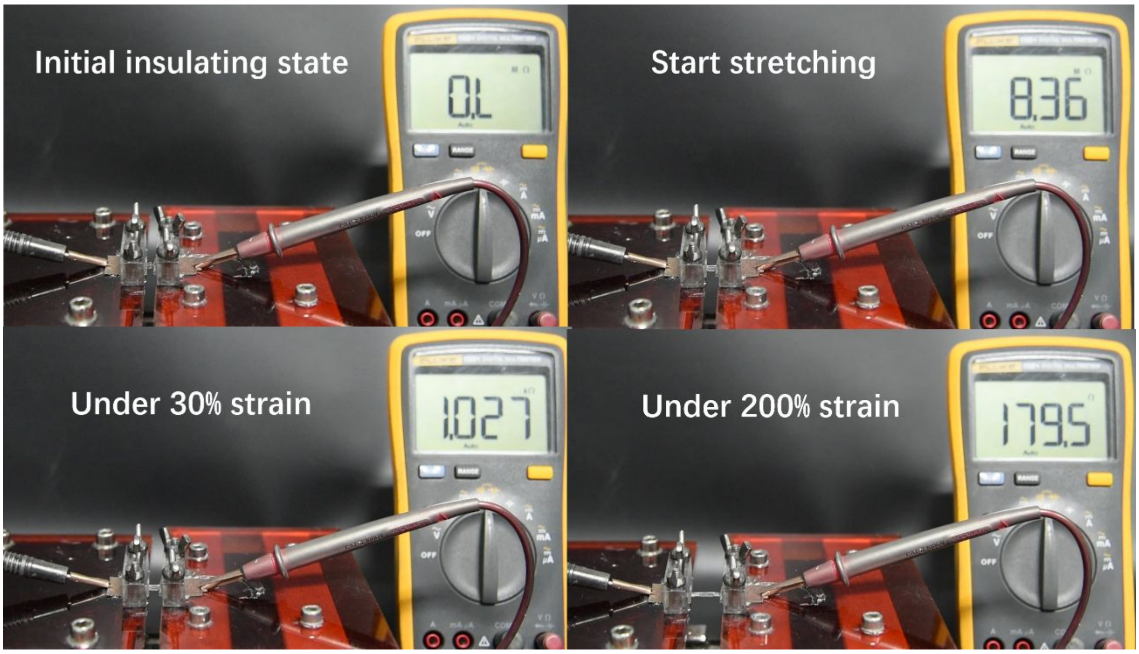

Figure S9. The strain-activated conductivity process for LMD-EG.

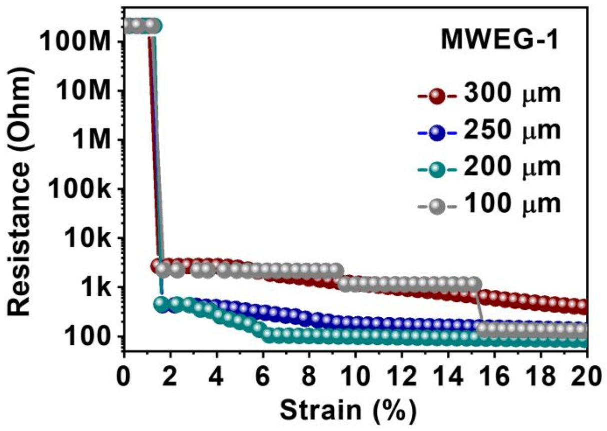

Figure S10. Resistance vs. the strain of LMD electrodes with a line width of 300, 250, 200, and 100 μm printed from MWEG-1.

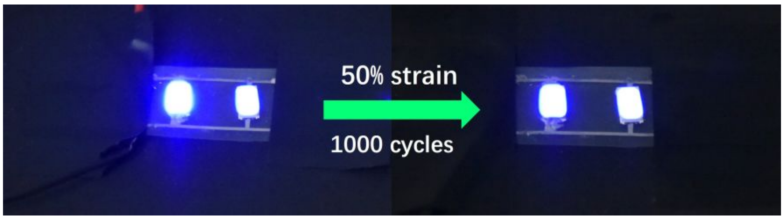

Figure S11. LEDs can work stably after the LMD-EG circuit was stretched to 50% strain over 1000 cycles.

Table S1. Summary of printing resolution of printed patterns based on liquid metal.

Materials	Resolution (μm)	Ref.
**MWEG-1**	**65**	**This work**
EGaIn/CNT	5	3
EGaIn	1.9	4
Oxidized GaIn	5000	5
EGaIn/PDMS	90	6
EGaIn/Ag flakes/SIS	200	7
EGaIn/SBS	1000	8
EGaIn/PVC	110	9

EGaIn/PDMS	500	10
EGaIn/Isopropanol	500	11
EGaIn	100	12
EGaIn	750	13
EGaIn	300	14

Table S2. Summary of electrical conductivity of stretchable conductors

Materials	Electrical Conductivity (S/cm)	Strain (%)	Ref.
**LMD-EG**	**15800**	**800**	**This work**
EGaIn/PDMS	24100	200	15
EGaIn/CPU	2479	700	16
EGaIn/PDMS	1370	50	10
Ag flakes/fluorine rubbers	6168	400	17
Ag flakes/fluorine rubbers	738	215	18
Ag particles/SBS	5450	140	19
Ag particles/CNT	5710	140	20
CNT/PDMS	2200	150	21
SWCNT/PDMS	1.08	100	22
Graphene/PDMS	0.72	60	23
PEDOT:PSS/SEBS	3600	600	24

Supporting movie 1: Strain-activated conductivity for LMD-EG.

Reference

(1) Boley, J. W.; White, E. L.; Kramer, R. K., Mechanically Sintered Gallium-Indium Nanoparticles. *Adv. Mater.* **2015**, *27*, 2355.

(2) Jia, Y.-G.; Jin, J.; Liu, S.; Ren, L.; Luo, J.; Zhu, X. X., Self-Healing Hydrogels of Low Molecular Weight Poly(viny-alcohol) Assembled by Host–Guest Recognition. *Biomacromolecules*. **2018**, *19*, 626.

(3) Park, Y. G.; Min, H.; Kim, H.; Zhexembekova, A.; Lee, C. Y.; Park, J. U., Three-Dimensional, High-Resolution Printing of Carbon Nanotube/Liquid Metal Composites with Mechanical and Electrical Reinforcement. *Nano Lett.* **2019**, *19*, 4866.

(4) Park, Y-G.; An H. S.; Kim, J-Y.; Park, J-U., High-Resolution, Reconfigurable Printing of Liquid Metals with Three-Dimensional Structures. *Sci. Adv*. **2019**, *5*, eaaw2844.

(5) Wang, X.; Fan, L.; Zhang, J.; Sun, X.; Chang, H.; Yuan, B.; Guo, R.; Duan, M.; Liu, J., Printed Conformable Liquid Metal e-Skin-Enabled Spatiotemporally Controlled Bioelectromagnetics for Wireless Multisite Tumor Therapy. *Adv. Funct. Mater*. **2019**, *29*, 1907063.

(6) Zhou, L-Y.; Fu, J-Z.; Gao, Q.; Zhao, P.; He, Y., All-Printed Flexible and Stretchable Electronics with Pressing or Freezing Activatable Liquid-Metal–Silicone Inks. *Adv. Funct. Mater*. **2020**, *30*, 1906683.

(7) Lopes, P. A.; Santos, B. C.; de Almeida, A. T.; Tabakoli, M., Reversible polymer-gel transition for ultra-stretchable chip-integrated circuits through self-soldering and self-coating and self-healing. *Nat. Commun.* **2021**, *12*, 4666.

(8) Ma, Z.; Huang, Q.; Xu, Q.; Zhuang, Q.; Zhao, X.; Yang, Y.; Qiu, H.; Yang, Z.; Wang, C.; Chai, Y.; Zheng, Z., Permeable Superelastic Liquid-Metal Fibre Mat Enables Biocompatible and Monolithic Stretchable Electronics. *Nat. Mater*. **2021**, *20*, 859.

(9) Wang, Q.; Yu, Y.; Yang, J.; Liu, J., Fast Fabrication of Flexible Functional Circuits Based on Liquid Metal Dual-Trans Printing. *Adv. Mater.* **2015**, *27*, 7109.

(10) Markvicka, E. J.; Bartlett, M. D.; Huang, X.; Majidi, C., An Autonomously Electrically Self-Healing Liquid Metal-Elastomer Composite for Robust Soft-Matter Robotics and Electronics. *Nat. Mater.* **2018**, *17*, 618.

(11) Mohammed, M. G.; Kramer, R., All-Printed Flexible and Stretchable Electronics. *Adv. Mater.* **2017**, *29*, 1604965.

(12) Zheng, Y.; He, Z-Z.; Yang, J.; Liu, J., Personal Electronics Printing via Tapping Mode Composite Liquid Metal Ink Delivery and Adhesion Mechanism. *Sci. Rep.* **2014**, *4*, 4588.

(13) Joshipura, I. D.; Ayers, H. R.; Castillo, G. A.; Ladd, C.; Tabor, C. E.; Adams, J. J.; Dickey, M. D., Patterning and Reversible Actuation of Liquid Gallium Alloys by Preventing Adhesion on Rough Surfaces. *ACS. Appl. Mater. Interfaces.* **2018**, *10*, 44686.

(14) Guo, R.; Tang, J.; Dong, S.; Lin, J.; Wang, H.; Liu, J.; Rao, W., One-Step Liquid Metal Transfer Printing: Toward Fabrication of Flexible Electronics on Wide Range of Substrates. *Adv. Mater. Technol*. **2018**, *3*, 1800265.

(15) Park, J.; Wang, S.; Li, M.; Ahn, C.; Hyun, J. K.; Kim, D. S.; Kim, D. K.; Rogers, J. A.; Huang, Y.; Jeon, S., Three-dimensional Nanonetworks for Giant Stretchability in Dielectrics and Conductors. *Nat. Commun.* **2012**, *3*, 916

(16) Park, S.; Thangavel, G.; Parida, K.; Li, S.; Lee, P. S., A Stretchable and Self-Healing Energy Storage Device Based on Mechanically and Electrically Restorative Liquid-Metal Particles and Carboxylated Polyurethane Composites. *Adv. Mater.* **2019**, *31*, 1805536.

(17) Matsuhisa, N.; Inoue, D.; Zalar, P.; Jin, H.; Matsuba, Y.; Itoh, A.; Yokota, T.; Hashizume, D.; Someya, T., Printable Elastic Conductors by In Situ Formation of Silver Nanoparticles from Silver Flakes. *Nat. Mater.* **2017**, *16*, 834.

(18) Matsuhisa, N.; Kaltenbrunner, M.; Yokota, T.; Jinno, H.; Kuribara, K.; Sekitani, T.; Someya, T., Printable Elastic Conductors with a High Conductivity for Electronic Textile Applications. *Nat. Commun*. **2015**, *6*, 7461.

(19) Park, M.; Im, J.; Shin, M.; Min, Y.; Park, J.; Cho, H.; Park, S.; Shim, M. B.; Jeon, S.; Chung, D. Y.; Bae, J.; Park, J.; Jeong, U.; Kim, K., Highly Stretchable Electric Circuits from a Composite Material of Silver Nanoparticles and Elastomeric Fibres. *Nat. Nanotech.* **2012**, *7*, 803.

(20) Chun, K. Y.; Oh, Y.; Rho, J.; Ahn, J. H.; Kim, Y. J.; Choi, H. R.; Baik, S., Highly Conductive, Printable and Stretchable Composite Films of Carbon Nanotubes and Silver. *Nat. Nanotech.* **2010**, *5*, 853.

(21) Lipomi, D. J.; Vosgueritchian, M.; Tee, B. C.; Hellstrom, S. L.; Lee, J. A.; Fox, C. H.; Bao, Z., Skin-Like Pressure And Strain Sensors Based On Transparent Elastic Films Of Carbon Nanotubes. *Nat. Nanotech.* **2011**, *6*, 788.

(22) Kim, K. H.; Vural, M.; Islam, M. F., Single-walled carbon nanotube aerogel-based elastic conductors. *Adv. Mater.* **2011**, *23*, 2865.

(23) Wang, Z.; Liu, X.; Shen, X.; Han, N. M.; Wu, Y.; Zheng, Q.; Jia, J.; Wang, N.; Kim, J. K., An Ultralight Graphene Honeycomb Sandwich for Stretchable Light‐Emitting Displays. *Adv. Funct. Mater.* **2018**, *28*, 1707043.

(24) Wang, Y.; Zhu, C.; Pfattner, R.; Yan, H.; Jin, L.; Chen, S.; Molina-Lopez F.; Lissel, F.; Liu, J.; Rabiah, N. I.; Chen, Z.; Chung, J. W.; Linder C.; Toney, M. F.; Murmann, B.; Bao, Z., A Highly Stretchable, Transparent and Conductive Polymer. *Sci. Adv.* **2017**, *3*, e1602076.
